# The nexus between appendicitis and chronic inflammatory bowel diseases: Unraveling an intriguing association

**DOI:** 10.1097/MD.0000000000038859

**Published:** 2024-10-11

**Authors:** Christoph Roderburg, Sven H. Loosen, Petra May, Kaneschka Yaqubi, Tom Luedde, Karel Kostev

**Affiliations:** a Department of Gastroenterology, Hepatology and Infectious Diseases, University Hospital Düsseldorf, Medical Faculty of Heinrich Heine University Düsseldorf, Düsseldorf, Germany; b Epidemiology, IQVIA, Frankfurt, Germany

**Keywords:** appendicitis, chronic inflammatory bowel diseases, colitis ulcerosa, Crohn Disease, epidemiology

## Abstract

Appendicitis is 1 of the most frequent diseases worldwide. In general, it is treated with appendectomy, which, in almost all cases, leads to the healing of the disease and averts acute complications. However, only limited data regarding long-term sequalae, including inflammatory bowel diseases following appendicitis are available. We therefore investigated the association between appendicitis and both Crohn disease (CD) and ulcerative colitis (UC). The present study included 23,991 patients with a history of appendicitis and 23,991 that did not have such a history. Patients were identified within the Disease Analyzer (IQVIA) database in Germany between 2010 and 2020. After a follow-up period of up to 10 years, 0.74% of patients with a history of appendicitis and 0.45% of those in the nonappendicitis cohort were diagnosed with CD (*P* < .001). Our regression analysis revealed a robust and statistically significant association between appendicitis and the incidence of CD in the entire study population (Hazard ratio: 1.82; 95% confidence interval [CI]: 1.31–2.53). Importantly, this association remained largely consistent across all age groups and both genders. In contrast, no statistically significant link was observed between appendicitis and the subsequent development of UC (Hazard ratio: 1.24; 95% CI: 0.90–1.71). The present study presents novel data from a large cohort of outpatients in Germany, providing strong evidence for an association between appendicitis and the development of CD (but not UC). These findings contribute to the existing body of literature and may facilitate the recognition of appendicitis as a risk factor for the development of chronic inflammatory bowel diseases.

## 1. Introduction

In the realm of gastrointestinal disorders, the etiology and pathogenesis of chronic inflammatory bowel diseases (IBD), including Crohn disease (CD) and ulcerative colitis (UC), have long remained a subject of intensive investigation and clinical scrutiny.^[1,2]^ The complex interplay of genetic, environmental, and immunological factors in the genesis of IBD has captivated the medical community for decades.^[1–3]^ In recent years, emerging evidence has pointed to a previously overlooked facet of this intricate puzzle – an association between prior appendicitis and an increased risk of developing chronic IBD.^[4]^

Appendicitis, representing 1 of the most frequent medical emergency worldwide, is frequently regarded as an isolated disease, with appendectomy providing definitive cure.^[5]^ However, increasing evidence has been generated on its long-term sequelae beyond its acute phase.^[6]^ Several studies have suggested that a history of appendicitis may exert a profound and enduring influence on the gastrointestinal milieu, potentially predisposing individuals to the onset or exacerbation of chronic diseases.^[6]^ In a recent systematic review and meta-analysis including 37 studies examining the surgical outcomes in terms of ileus, cancer, fertility, incisional hernia and IBD, the prevalence of CD was higher, and the prevalence of UC was lower after appendectomy than in controls.^[7]^ This burgeoning area of investigation raises critical questions about the mechanisms underpinning this association, the clinical implications for patients with a history of appendicitis, and the broader implications for our understanding of the pathogenesis of chronic IBD.

By examining the link between appendectomy and chronic IBD, we hope to provide a better insight into the pathophysiology of IBD and pave the way for novel approaches in the clinical management of patients with a history of this seemingly innocuous but potentially far-reaching condition.

## 2. Methods

### 2.1. Database

This retrospective cohort study was based on data from the IQVIA Disease Analyzer, which has been recently described in detail (e.g.^[8]^) and has been extensively been used to study the epidemiology of gastrointestinal pathologies.^[9,10]^

### 2.2. Study population

**Figure 1.**
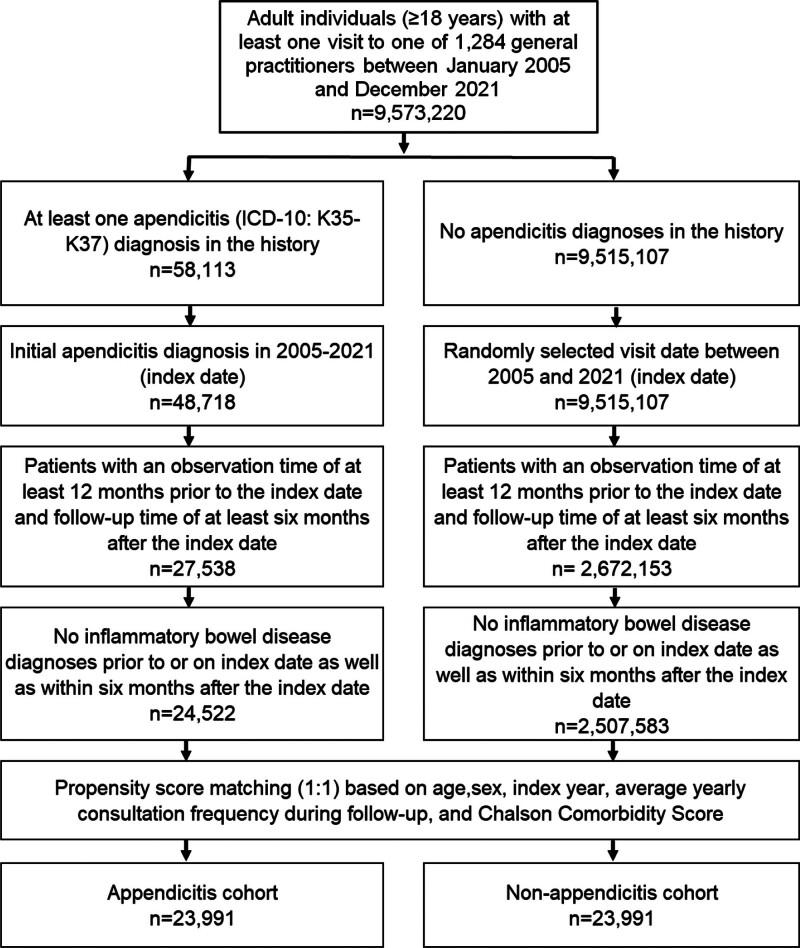
Selection of study patients.

This study includes adult patients (≥ 18 years) with an initial diagnosis of appendicitis (ICD-10: K35–K37) in 1284 general practices in Germany between January 2005 and December 2021 (index date, see Fig. 1). Further inclusion criterium was an observation time of at least 12 months prior to the index date and a follow-up time of at least 6 months after the index date. Patients diagnosed with IBD (ICD-10: K50–K52) prior to or on index date as well as within 6 months after the index date were excluded. We excluded patients with IBD diagnoses within 6 months after index date to avoid the possible misclassification of IBD as appendicitis in the case where appendicitis-like symptoms occur as first symptoms of IBD. After applying similar inclusion criteria, individuals without appendicitis were matched to appendicitis patients using propensity score matching (1:1) based on age, sex, index year, average yearly consultation frequency during follow-up, and Charlson Comorbidity Index (CCI). The CCI includes a wide range of comorbidities (macrovascular diseases, pulmonary diseases, gastrointestinal, liver, and renal diseases, diabetes, Acquired Immunodeficiency Syndrome [AIDS], and others).^[11]^ For the nonappendicitis cohort, the index date was that of a randomly selected visit between January 2005 and December 2021 (Fig. 1).

### 2.3. Study outcomes and statistical analyses

The outcomes of the study were the initial diagnoses of CD (ICD-10: K50) and UC within 10 years following the index date as a function of appendicitis. Kaplan–Meier curves were compared using the log-rank test. Univariable Cox regression analyses were conducted to assess the association between appendicitis and CD and UC in the total cohort as well as in 3 age groups, women and men separately. As 2 comparisons were made (CD, UC), we considered a *P*-value of <0.025 as statistically significant. Analyses were carried out using SAS version 9.4 (SAS Institute, Cary, NC, USA).

## 3. Results

### 3.1. Basic characteristics of the study sample

The present study included 23,991 individuals with appendicitis and 23,991 without appendicitis. The basic characteristics of the study patients are displayed in Table 1. In summary, mean age was 43.0 (SD: 18.6) years, while 55.8% of patients were female. On average, patients visited physicians 6.2 times per year during the follow-up, with no statistically significant differences between both patient cohorts. Of note, the mean CCI was 1.2 in both groups. Most patients were included between 2017 and 2021 (index years).

**Table 1:** Baseline characteristics of the study sample (after 1:1 matching).

Variable	Proportion among appendicitis patients (N, %) N = 23,991	Proportion among nonappendicitis patients (N, %) N = 23,991	*P* value
Age (Mean, SD)	43.0 (18.6)	43.0 (18.6)	1.000
Age 18 to 30	8030 (33.5)	8030 (33.5)	1.000
Age 31 to 50	7673 (32.0)	7673 (32.0)	
Age > 50	8288 (34.6)	8288 (34.6)	
Female	13,380 (55.8)	13,378 (55.8)	0.98.5
Male	10,611 (44.2)	10,613 (44.2)	
Number of physician visits per year during the follow-up (Mean, SD)	6.2 (3.9)	6.2 (3.9)	1.000
Charlson Comorbidity Score (CCS)	1.2	1.2	1.000
CCS 0	10,143 (42.3)	10,145 (42.3)	1.000
CCS 1	6965 (29.0)	6969 (29.0)	
CCS 2	3069 (12.8)	3067 (12.8)	
CCS 3	1652 (6.9)	1651 (6.9)	
CCS >3	2162 (9.0)	2159 (9.0)	
Index year 2005 to 2008	2601 (10.8)	2602 (10.8)	1.000
Index year 2009 to 2012	4480 (18.7)	4483 (18.7)	
Index year 2013 to 2016	6316 (26.3)	6315 (26.3)	
Index year 2017 to 2021	10,594 (44.2)	10,591 (44.2)	

Proportions of patients given in N, %, unless otherwise indicated. SD: standard deviation.

### 3.2. Association of appendicitis with subsequent CD

**Figure 2.**
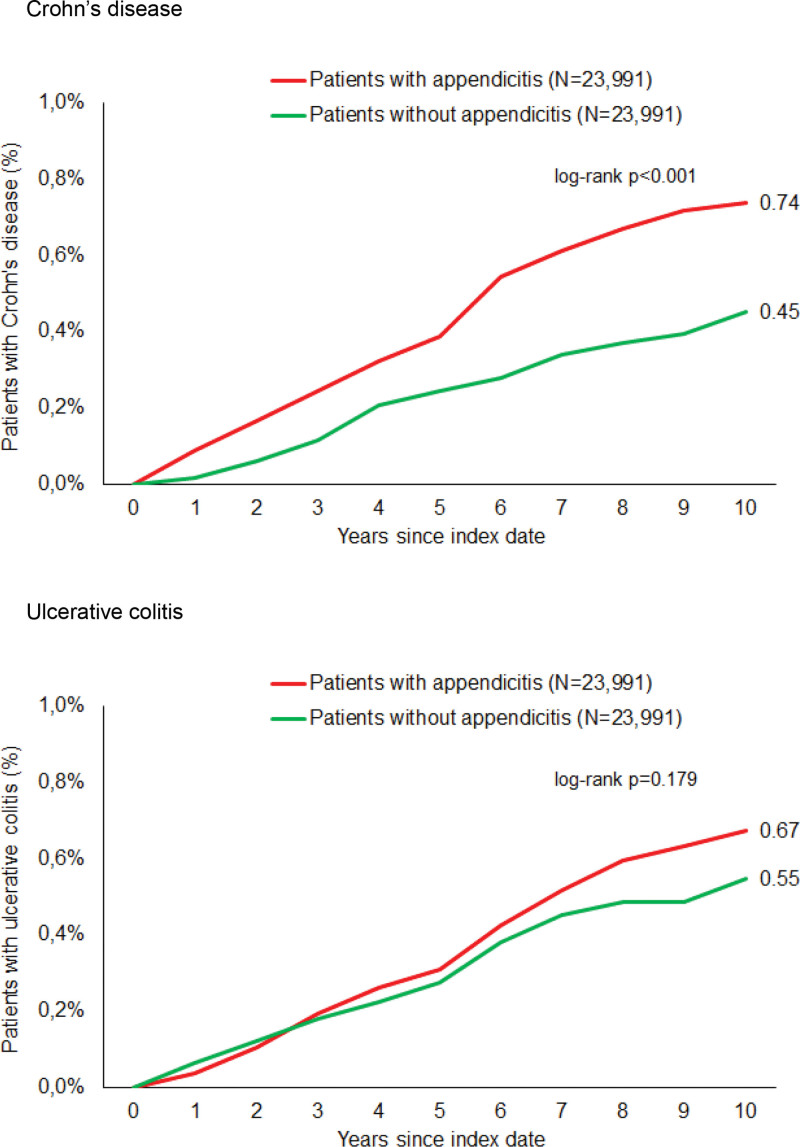
Cumulative incidence of Crohn disease and ulcerative colitis in patients with and without appendicitis.

After a follow-up period of up to 10 years, 0.74% of patients with a history of appendicitis, but only 0.45% of patients from the nonappendicitis cohort, were diagnosed with CD (*P* < .001, Fig. 2). In regression analysis, a robust and statistically significant association between appendicitis and subsequent CD within the total population (Hazard ratio: 1.82; 95% confidence interval [CI]: 1.31–2.53) was observed. In the age-stratified analyses, the association was most pronounced within the age group of 31 to 50 (HR: 2.10; 95% CI: 1.18–3.77), while the effect was not significant among patients aged over 60 (HR: 1.35; 95% CI: 0.71–2.59). Both incidence and prevalence of CD largely vary between male and female. Of note, the association between appendicitis and CD was similar in women (HR: 1.85; 95% CI: 1.21–2.84) and men (HR: 1.76; 95% CI: 1.04–2.97). In the case of men, the *P*-value (.035) did not meet the predefined significance level (Table 2).

**Table 2:** Association between appendicitis and subsequent Crohn disease and ulcerative colitis diagnoses in patients followed in general practices in Germany (univariable Cox regression models).

Patient group	CD		UC	
	Hazard ratio (95% CI)	*P* value	Hazard ratio (95% CI)	*P* value
Total	1.82 (1.31–2.53)	<.001	1.24 (0.90–1.71)	.180
Age 18 to 30	1.93 (1.16–3.24)	.012	1.10 (0.59–2.03)	.771
Age 31 to 50	2.10 (1.18–3.77)	.012	1.53 (0.86–2.73)	.153
Age >60	1.35 (0.71–2.59)	.364	1.16 (0.71–1.88)	.564
Female	1.85 (1.21–2.84)	.005	1.36 (0.89–2.10)	.158
Male	1.76 (1.04–2.97)	.035	1.11 (0.69–1.79)	.673

### 3.3. Association of appendicitis with subsequent UC

The cumulative incidence of UC was 0.67% in the appendicitis cohort and 0.55% in the nonappendicitis cohort (*P* = .179) (Fig. 2). In the regression analysis, there was no significant association between appendicitis and subsequent UC (HR: 1.24; 95% CI: 0.90–1.71). In addition, no significant associations were observed within the age- and sex-stratified analyses (Table 2).

## 4. Discussion

Examining a total of 50,000 patients (23,991 individuals with appendectomy and 23,991 without appendectomy), we provide evidence that prior appendectomy is associated with the development of CD, while no such association is observed with UC. These findings represent the first data from German outpatients on this link, paving the way to further establish appendicitis as a disease associated with a subsequent CED.

The present data are in line to recent findings. A previous systematic review and meta-analysis demonstrated that appendectomy increased the incidence of CD while reducing the rate of UC. Similar outcomes were described by Kaplan et al^[12]^ as well as by Gardenbroek et al,^[13]^ underlining the reliability of our data from a large cohort of patients treated in over 3000 German outpatient units. However, it is noteworthy that none of these trials provided evidence on a causal relationship between appendicitis and the development of chronic IBD. In line, it is suspected that the higher prevalence of CD, especially shortly after appendectomy, may be attributed to difficulties in diagnosing incipient CD.^[14]^ In this context, it is important to note that patients diagnosed with IBD prior to or on index date as well as within 6 months after the index date were excluded. Moreover, Kaplan–Meier curves show that the effect of appendicitis on the development of CD is more prominent in the long-term perspective rather than in close temporal proximity to the diagnosis of appendicitis. On a mechanistic level, the appendix represents an organ with a large number of diverse immune cells that is deeply integrated into the local and systemic immune response against a range of antigens.^[15]^ Thus, it remains plausible that an appendectomy can impact the development of CD and UC as inflammatory diseases, potentially triggered by an altered immunological milieu. Further research is needed to better understand a potential causal link between both diseases.

Appendectomy is a routinely performed surgical procedure,^[16]^ and like any surgical intervention, it carries potential risks and complications, both in the short term and over the long term. Short-term complications of appendectomy, such as infections and intra-abdominal abscesses, are generally manageable and do not pose significant concern in routine clinical practice. In contrast, the long-term complications are only poorly understood. Recent studies have examined the effects of appendix removal on various aspects, including ileus, incisional hernia, cancer risk, fertility, and overall mortality. It is noteworthy that, while there is an increased incidence of CD following appendectomy, no other significant long-term consequences have been definitely established.

A major strength of the presented analysis is the large sample size. Furthermore, the database used for this analysis has been widely been used to study the epidemiology of gastrointestinal pathologies and proved to be representative in this context.^[8]^ Importantly, the fact that the database has previously been used to analyze inflammatory as well as malignant diseases^[9,10,17–19]^, adds to the reliability of the data presented. Of course, it is essential to acknowledge significant limitations that should be taken into account when interpreting our study’s outcomes. Diagnosed were coded using ICD-10, potentially introducing misclassifications and both over- and undercoding of specific pathologies. This analysis focused on examining the relationship between appendicitis and chronic IBD and adjusted for age and gender as confounding factors. Unfortunately, many data elements necessary for further analysis were lacking. These included the patients´ socioeconomic status, genetic predisposition and different other conditions that may predispose for CD and UC. While our study’s results align with those of prior research, it is important to note that associations but not causal relationships were shown. Nevertheless, we are the first to provide data in the respective context from German medical practices.

In conclusion, our results provide compelling evidence that prior appendicitis is associated with the development of CD but not with UC. Notably, these observations were consistent across different age groups and genders. Thus, appendicitis may represent a previously unrecognized factor that could be employed for stratification of patients according to their risk for development of CD. Further research is needed to better understand the detailed mechanism linking both diseases.

## Author contributions

**Conceptualization:** Christoph Roderburg, Sven H. Loosen, Tom Luedde, Karel Kostev.

**Writing – original draft:** Christoph Roderburg, Sven H. Loosen, Kaneschka Yaqubi, Petra May, Tom Luedde, Karel Kostev.

**Writing – review & editing:** Christoph Roderburg, Sven H. Loosen, Kaneschka Yaqubi, Petra May, Tom Luedde, Karel Kostev.

**Supervision:** Sven H. Loosen, Karel Kostev.

**Data curation:** Karel Kostev.

**Formal analysis:** Karel Kostev.

**Project administration:** Karel Kostev.

**Visualization:** Karel Kostev.

## References

[1] Abraham C Cho JH. Inflammatory bowel disease. N Engl J Med. 2009;361:2066–78. 19923578 10.1056/NEJMra0804647 PMC3491806

[2] Zhao M Gönczi L Lakatos PL Burisch J. The burden of inflammatory bowel disease in Europe in 2020. J Crohns Colitis. 2021;15:1573–87. 33582812 10.1093/ecco-jcc/jjab029

[3] Alatab S Sepanlou SG Ikuta K. The global, regional, and national burden of inflammatory bowel disease in 195 countries and territories, 1990–2017: a systematic analysis for the Global Burden of Disease Study 2017. Lancet Gastroenterol Hepatol. 2020;5:17–30. 31648971 10.1016/S2468-1253(19)30333-4 PMC7026709

[4] Arjomand Fard N Armstrong H Perry T Wine E. Appendix and ulcerative colitis: a key to explaining the pathogenesis and directing novel therapies? Inflamm Bowel Dis. 2023;29:151–60. 35749298 10.1093/ibd/izac106 PMC9825289

[5] Bhangu A Søreide K Di Saverio S Assarsson JH Drake FT. Acute appendicitis: modern understanding of pathogenesis, diagnosis, and management. Lancet (London, England). 2015;386:1278–87. 26460662 10.1016/S0140-6736(15)00275-5

[6] Lee S Jang EJ Jo J Park SJ Ryu HG. Long-term impacts of appendectomy associated with increased incidence of inflammatory bowel disease, infection, and colorectal cancer. Int J Colorectal Dis. 2021;36:1643–52. 33594506 10.1007/s00384-021-03886-x

[7] Rasmussen T Fonnes S Rosenberg J. Long-Term complications of appendectomy: a systematic review. Scand J Surg. 2018;107:189–96. 29764306 10.1177/1457496918772379

[8] Rathmann W Bongaerts B Carius HJ Kruppert S Kostev K. Basic characteristics and representativeness of the German Disease Analyzer database. Int J Clin Pharmacol Ther. 2018;56:459–66. 30168417

[9] Zingel R Bohlken J Kostev K. Association between inflammatory bowel disease and dementia: a retrospective cohort study. J Alzheimer's Dis. 2021;80:1471–8. 33720902 10.3233/JAD-210103

[10] Loosen SH Yaqubi K May P. Association between inflammatory bowel disease and subsequent development of restless legs syndrome and Parkinson’s disease: a retrospective cohort study of 35,988 primary care patients in Germany. Life (Basel). 2023;13:897. 37109426 10.3390/life13040897 PMC10145108

[11] Quan H Sundararajan V Halfon P. Coding algorithms for defining comorbidities in ICD-9-CM and ICD-10 administrative data. Med Care. 2005;43:1130–9. 16224307 10.1097/01.mlr.0000182534.19832.83

[12] Kaplan GG Jackson T Sands BE Frisch M Andersson RE Korzenik J. The risk of developing Crohn’s disease after an appendectomy: a meta-analysis. Am J Gastroenterol. 2008;103:2925–31. 18775018 10.1111/j.1572-0241.2008.02118.x

[13] Gardenbroek TJ Eshuis EJ Ponsioen CIJ Ubbink DT D’Haens GRAM Bemelman WA. The effect of appendectomy on the course of ulcerative colitis: a systematic review. Colorectal Dis. 2012;14:545–53. 21689293 10.1111/j.1463-1318.2011.02600.x

[14] Frisch M Johansen C Mellemkjær L. Appendectomy and subsequent risk of inflammatory bowel diseases. Surgery. 2001;130:36–43. 11436010 10.1067/msy.2001.115362

[15] Kooij IA Sahami S Meijer SL Buskens CJ te Velde AA. The immunology of the vermiform appendix: a review of the literature. Clin Exp Immunol. 2016;186:1–9. 27271818 10.1111/cei.12821 PMC5011360

[16] Ferris M Quan S Kaplan BS. The global incidence of appendicitis. Ann Surg. 2017;266:237–41. 28288060 10.1097/SLA.0000000000002188

[17] Tanislav C Trommer K Labenz C Kostev K. Inflammatory bowel disease as a precondition for stroke or TIA: a matter of Crohn’s disease rather than ulcerative colitis. J Stroke Cerebrovasc Dis. 2021;30:105787. 33865232 10.1016/j.jstrokecerebrovasdis.2021.105787

[18] Loosen SH Kostev K Keitel V Tacke F Roderburg C Luedde T. An elevated FIB-4 score predicts liver cancer development: a longitudinal analysis from 29,999 NAFLD patients. J Hepatol. 2021;76:247–8. 34520785 10.1016/j.jhep.2021.08.030

[19] Loosen SH Kostev K Schöler D. Infectious mononucleosis is associated with an increased incidence of Crohn’s disease: results from a cohort study of 31 862 outpatients in Germany. Eur J Gastroenterol Hepatol. 2023;35:255–60. 36708295 10.1097/MEG.0000000000002505

